# Implementation of a Novel, Intensive, Two-Week Simulation Rotation for Pediatric Residents to Teach Crisis Resource Management Skills

**DOI:** 10.7759/cureus.94582

**Published:** 2025-10-14

**Authors:** Lorel R Huber, Robert Bishop, Shannon M Flood, Ryan Good, Christopher Nichols, Kristen Miller, Kathryn Walsh

**Affiliations:** 1 Pediatric Critical Care Medicine, University of Colorado, Aurora, USA; 2 Pediatric Cardiology, University of Colorado, Aurora, USA; 3 Pediatric Emergency Medicine, University of Colorado, Aurora, USA; 4 Pediatric Anesthesiology, University of Colorado, Aurora, USA; 5 Pediatrics, University of Colorado, Aurora, USA

**Keywords:** crisis resource management skills, leader, pediatric residency education, simulation in medical education, simulation rotation

## Abstract

Background

Pediatric residents must be competent in leading resuscitations, but have few clinical opportunities to develop their crisis resource management (CRM) skills. Simulation-based medical education (SBME), including CRM training, can supplement resident education where clinical experience is lacking.

Objectives

To develop and evaluate the feasibility, acceptability, and effectiveness of an SBME rotation for pediatric residents to improve competency and confidence in CRM skills.

Methodology

We designed a 10-day rotation with 31 unique simulations for residents interested in developing their CRM skills. A pre- and post-course survey evaluated the acceptability and effectiveness of the rotation. To assess leadership skills, a core component of CRM, we used the Concise Assessment of Leader Management (CALM) tool.

Results

Over six rotations, 38 residents participated, and 27 (71%) completed both the pre- and post-surveys. All 27 residents would recommend the rotation to other residents. After completing the course, the residents reported improvement in confidence (median (interquartile range, IQR): 2 (2, 3) vs. 4 (3, 4), *P*-value < 0.0001, *n *= 27) and capability (median (IQR): 3 (2, 3) vs. 4 (4, 4), *P*-value < 0.0001, *n *= 27) when approaching a patient requiring resuscitation. CALM scores improved for the residents in their last simulation compared to their first simulation (median (IQR): 52.50 (49.25, 55.00) vs. 30.50 (20.75, 35.50), *P*-value < 0.0001, *n* = 16), indicating an improvement in leadership.

Conclusions

We developed a novel, intensive, simulation-based rotation for pediatric residents that was feasible, acceptable, and improved residents’ capability, confidence, and leadership skills when approaching a patient resuscitation.

## Introduction

Per the 2025 Accreditation Council for Graduate Medical Education (ACGME) requirements, pediatric residents must complete training, maintain certification, and achieve competence in advanced life support skills in pediatrics and neonates [1]. In addition to resuscitation skills, residents need to develop effective communication and leadership skills [1]. Effectiveness in these skills is encompassed in crisis resource management (CRM) training. CRM is a set of cognitive and interpersonal behaviors that contribute to optimal resuscitation team performance and includes leadership, communication, anticipation and planning, resource utilization, workload distribution, situational awareness, triage and prioritization, and management of disruptions [2]. Due to the rarity of pediatric resuscitations and decreased intensive care unit time, pediatric residents have limited experience with CRM skills in clinical practice and subsequently lack the knowledge and skills required to be competent leaders at resuscitations and code events [3,4].

It is important to explore simulation-based medical education (SBME) as an adjunct in pediatric resident CRM training. While other teaching methods, such as the flipped classroom method, problem-based learning, and specialized study modules, are often used in medical education, the use of SBME has the benefit of providing an opportunity for trainees to practice CRM techniques and receive direct, expert feedback without the risk of harm to patients [5]. Simulation can improve pediatric residents’ CRM skills, including leadership skills, resuscitation skills, team performance, and supplement existing clinical gaps [6-13]. A modified Delphi study on the use of simulation for pediatric residents highlighted resuscitation and leadership skills as important curricular content for teaching pediatric residents [14]. Despite the benefits of SBME for teaching pediatric residents CRM skills, there is limited literature on the existence or efficacy of an intensive simulation rotation. Utilizing experiential learning as a conceptual framework, we designed and implemented an intensive, simulation-based rotation for pediatric residents. In this report, we aim to describe the feasibility, acceptability, and effectiveness of a simulation-based rotation for pediatric residents to improve competency and confidence in CRM skills, including leadership and resuscitation. Parts of this article were previously presented as a meeting abstract at the 2023 Association of Pediatric Program Directors Meeting on March 29, 2023, and at the 2023 Pediatric Academic Societies Meeting on April 29, 2023.

## Materials and methods

The simulation course was developed for a large pediatric residency program by two authors (LH and KW) and reviewed by the study team and leaders from the pediatric residency program. We conducted a prospective, observational, cohort study to describe the feasibility, acceptability, and effectiveness of a simulation-based rotation for pediatric residents. The study was deemed exempt by the Colorado Multiple Institutional Review Board (IRB number 21-2846, exempt 3/1/2021), and participants were provided with a postcard consent.

Curriculum design

**Figure 1:**
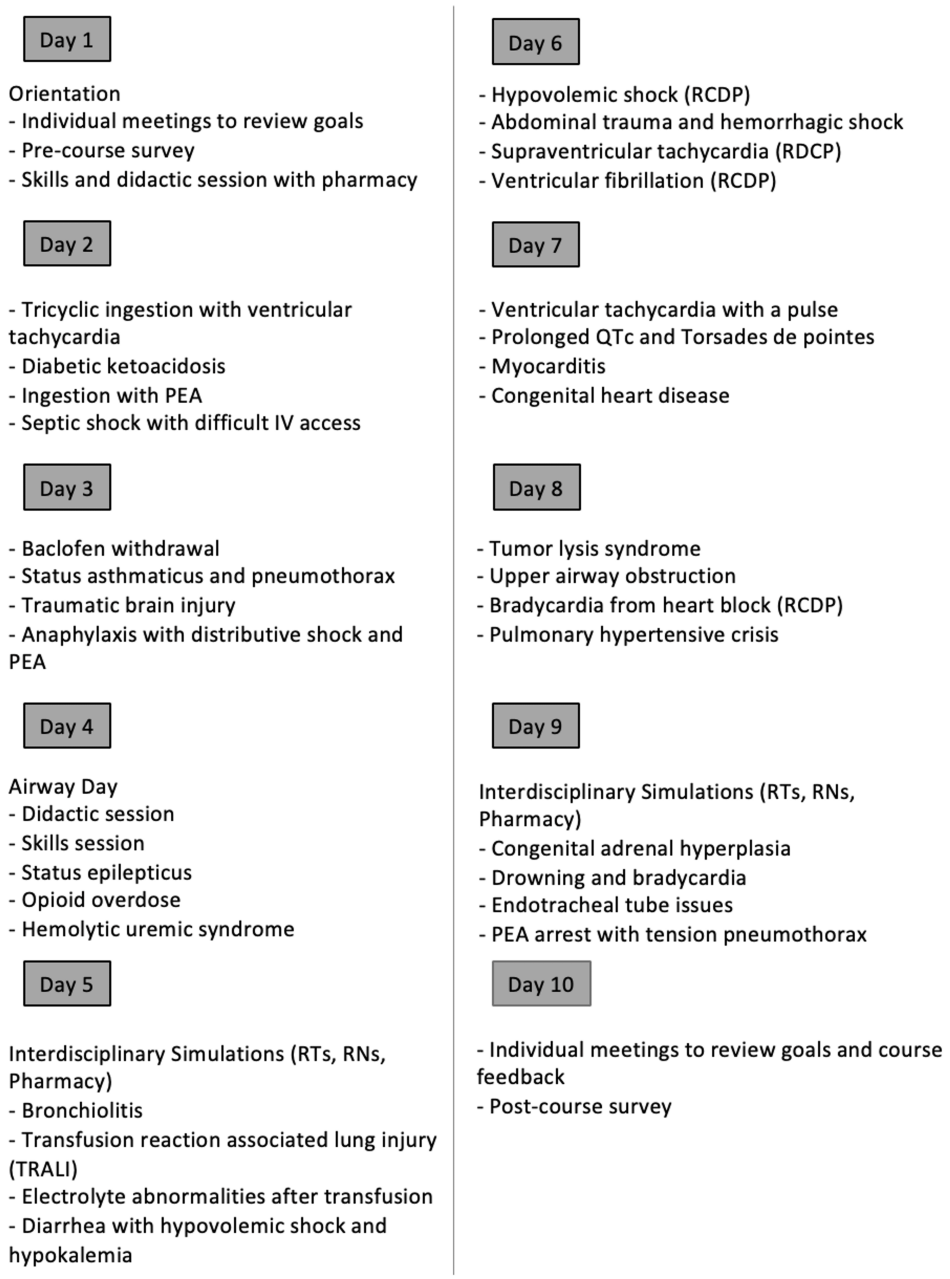
Sample simulation rotation schedule. RTs, respiratory therapists; RNs, registered nurses; PEA, pulseless electrical activity; RCDP, rapid cycle deliberate practice Image credit: Lorel Huber.

The study team used Kern’s six steps of curriculum development to design this course with the goal of improving competency and confidence in CRM skills, including leadership and resuscitation skills [15]. Two course directors designed a 10-day rotation for senior pediatric residents that includes 31 high-fidelity simulations and is offered twice per year for up to seven residents per rotation. Residents self-select into the rotation. Rotation schedule and logistics are listed in Figure 1.

Simulations were conducted at two institutions using high-fidelity mannequins and Laerdal simulation software. Teaching activities on most days lasted four hours. Most simulations are debriefed using the “Debriefing with Good Judgment” model, with several Rapid Cycle Deliberate Practice (RCDP) sessions [8,16]. The course directors assign the leaders of each simulation to ensure all residents serve in the leader role with similar frequency. Course directors meet individually with each resident on the first and last day of the rotation to review individual goals.

Program implementation and evaluation

Course evaluation was collected through an anonymous pre- and post-course survey (Appendices A-B) conducted from 2021 to 2024. This survey includes demographic information, resuscitation experience, and measures of rotation acceptability and effectiveness. Inclusion criteria for participants were second and third-year pediatric or combined residents within our program. There were no exclusion criteria.

Feasibility

To evaluate feasibility, we identified barriers and required resources for rotation implementation. A list of required resources is located in Table 1. 

**Table 1:** List of course development requirements.

Personnel	Equipment/Materials	Preparation	Time
Course directors	Simulation equipment and mannequins	Facilitator training	Scheduling time in simulation center
Course facilitators	Simulation software	Defining course objectives	Time of course directors and facilitators
Resident participation	Simulation scenarios	Support from institution/program	
Interdisciplinary participants		Support from simulation center	
Simulation center staff members		Coordination of residency schedules	
		Coordination of facilitators and interdisciplinary participants	
		Course evaluation and feedback form	

Acceptability

For the post-survey, we anonymously asked residents if they liked the course and if they would recommend it to other residents. They also provided free response feedback. Course directors met with residents after the course to obtain additional feedback.

Effectiveness

With the pre- and post-surveys, we utilized a five-point (1 = not at all, 5 = extremely) Likert scale for self-perceived confidence, capability, and anxiety when approaching a patient resuscitation. The survey included a five-point Likert scale for self-perceived confidence of various resuscitation skills (intubation, bag-mask ventilation, etc.). For three rotations, we evaluated the residents in the leader role using the Concise Assessment of Leader Management (CALM) instrument to assess improvement in leadership, a major component of CRM, at the beginning and end of the course [13]. The CALM instrument is an assessment instrument that can facilitate formative feedback to leaders of simulated pediatric resuscitation [13]. This tool has 15 four-point Likert scale items, one 2-point behavioral item, and evaluates leadership, communication, team management, and medical management. When scoring the 16 items, the maximum score is 62 [17]. A higher score indicates superior leadership. Course directors had prior experience using the CALM tool and scored residents leading their first and last simulation in real time.

Statistical methods

Variables were summarized using frequency and percentage (*n*, %) or median and interquartile range (IQR). We used a Wilcoxon signed-rank test to compare pre- versus post-scores on survey responses and the CALM tool. Analysis was performed using R version 3.4.1.

## Results

Thirty-eight residents completed the rotation. From 2021 to 2024, we conducted six rotations, with a total of 38 residents. Thirty-five (92%) residents completed the pre-survey, and 27 (71%) completed both the pre-/post-survey. A condensed summary of pre-survey results is listed in Table 2. 

**Table 2:** Resident demographics and resuscitation experience. PALS, Pediatric Advanced Life Support

	Respondents, *n* (%)
Year in pediatric residency (*n* = 35)	
Second-year residents	9 (26)
Third-year residents	26 (74)
Planned subspecialty (*n* = 35)	
Pediatric Critical Care Medicine	8 (23)
Pediatric Emergency Medicine	7 (20)
Pediatric Hospital Medicine	7 (20)
Neonatology	5 (14)
Pediatric Cardiology	2 (6)
Pediatric Gastroenterology	2 (6)
Pediatric Primary Care	2 (6)
Other	2 (6)
How many actual resuscitations have you been to in residency (excluding delivery resuscitations)? (*n* = 35)	
0	1 (3)
1-2	10 (29)
3-5	12 (34)
6-9	7 (20)
>9	5 (14)
How many simulated pediatric resuscitations have you participated in during residency? (*n* = 35)	
0	0 (0)
1-2	1 (3)
3-5	5 (14)
6-9	16 (46)
> 9	13 (37)
How important is it that you receive training using simulation modalities (low-fidelity, high-fidelity, and procedural) to prepare you to lead resuscitations and demonstrate competency in bag-mask ventilation, chest compressions, intubation, use of a ZOLL defibrillator, and PALS algorithms? (*n* = 35)	
Not at all important	0 (0)
A little important	0 (0)
Somewhat important	0 (0)
Pretty important	3 (9)
Very important	32 (91)

Feasibility

Course development required obtaining support from the pediatric residency program, as well as support from two institutions where simulations are held. Both locations utilized high-fidelity simulation mannequins and software that had already been purchased, so no additional costs were incurred. We recruited other formally trained simulationists to help facilitate simulations. Two course directors had a time commitment of at least 40 hours each over two weeks. Scheduling of the rotation was done by the course directors and required balancing residents’ other commitments, including clinics and other educational requirements.

Acceptability

All residents (27/27, 100%) responded that they would *definitely* recommend and *loved* the rotation. Specific comments from the post-survey and feedback meetings include: “The rotation has been great! dense, high-quality learning. The most educational elective that I’ve done in residency. The best rotation of all of residency. Time was high yield. Truly the most valuable two weeks of my residency experience so far.”

Effectiveness

**Figure 2:**
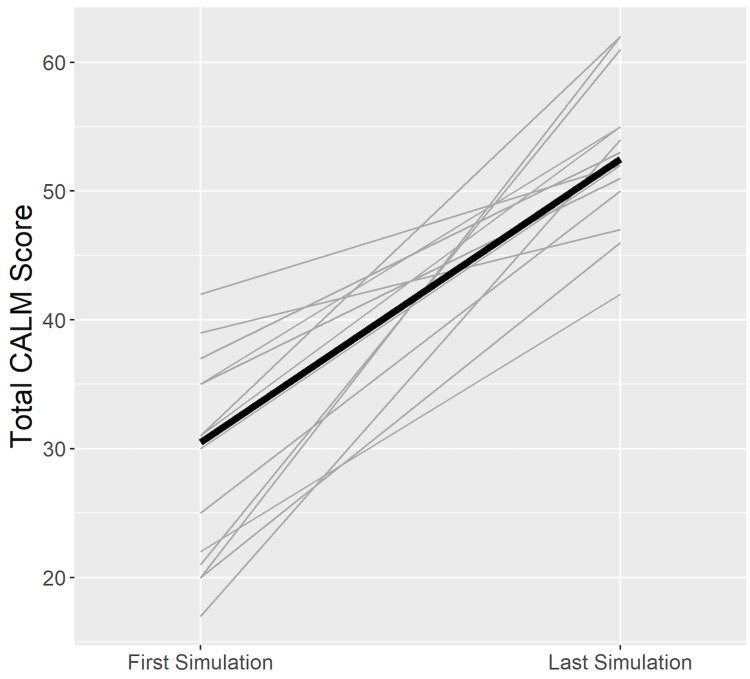
CALM scores of pediatric residents. Residents in three rotations were evaluated while leading their first and last simulations using the Concise Assessment of Leader Management (CALM) instrument. Total possible score of 62, with higher scores indicating better leader strength. The light gray lines represent individual residents, and the solid black line is the change in the median of the group.

Sixteen residents were evaluated using the CALM tool. There was significant improvement in the CALM score on the residents’ last simulation as compared to their first simulation (median 52.50, interquartile range (IQR): 49.25-55 vs. median of 30.50, IQR: 20.75-35.50, *P*-value < 0.0001 by Wilcoxon signed-rank test; *n *= 16), indicating improvement in leadership ability at the end of the course (Figure 2).

**Figure 3:**
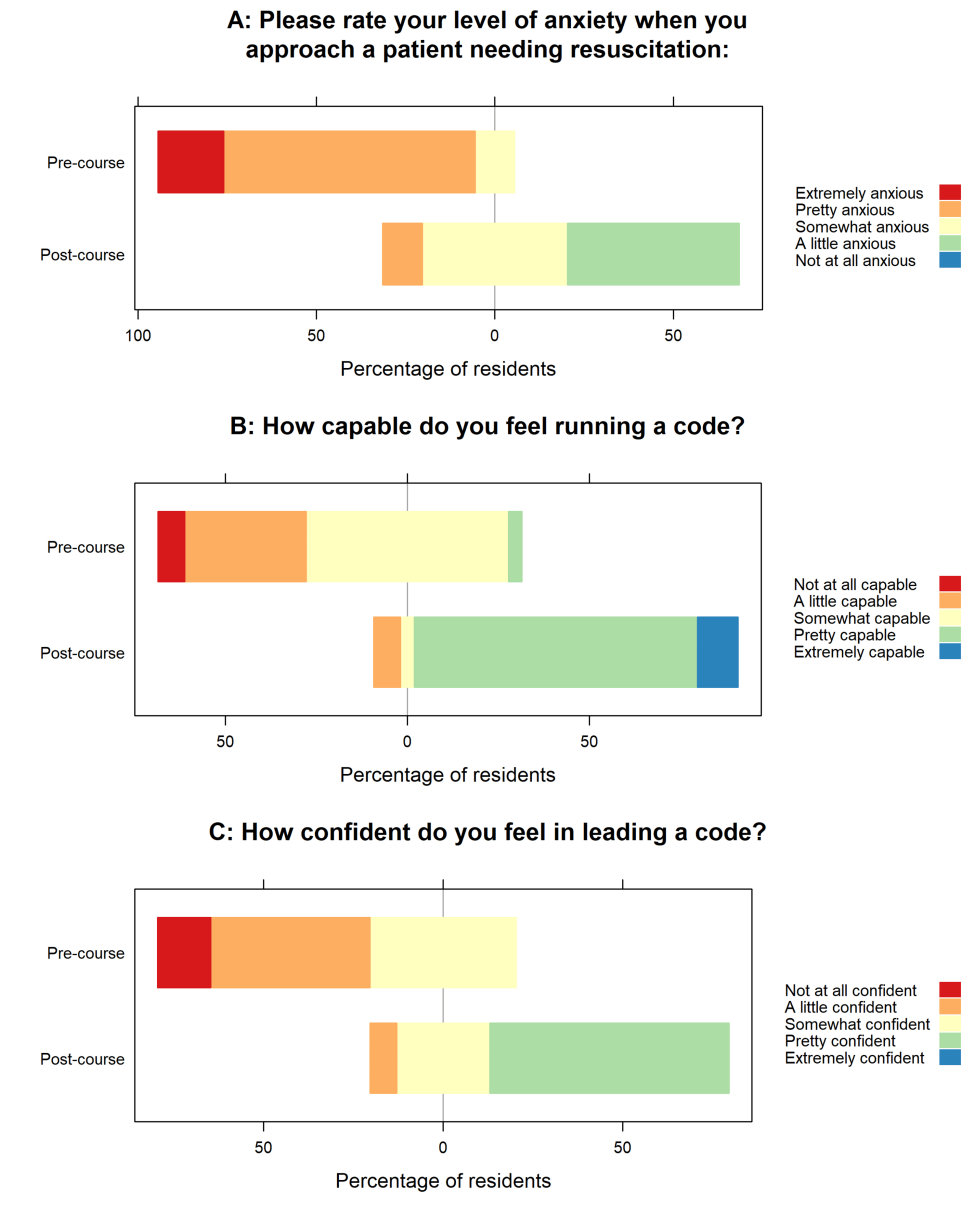
Likert scale results from pediatric residents on self-reported anxiety, capability, and confidence when approaching a patient requiring resuscitation. (A) Level of anxiety. Residents reported decreased anxiety when approaching a patient requiring resuscitation, with a median of 4 (pretty anxious, 4, 4) before versus 3 (somewhat anxious, 2, 3) after the course (*P* < 0.0001; *n* = 27). (B) Resident capability in running a code. Residents reported a significant improvement in self-perceived capability, with a median of 3 (somewhat capable, 2, 3) before versus 4 (pretty capable, 4, 4) after the course (*P* < 0.0001; *n* = 27). (C) Resident confidence in leading a code. Residents reported a significant increase in self-perceived confidence, with a median of 2 (a little confident, 2, 3) before versus 4 (pretty confident, 3, 4) after the course (*P* < 0.0001; *n* = 27).

Residents reported significant improvement in self-perceived confidence (median (IQR): 2 (2, 3) vs. 4 (3, 4), *P*-value < 0.0001, *n *= 27) and capability (median (IQR): 3 (2, 3) vs. 4 (4, 4), *P*-value < 0.0001, *n *= 27) when approaching a patient requiring resuscitation before and after the course. Residents reported decreased anxiety (median (IQR): 4 (4, 4) vs. 3 (2, 3), *P*-value < 0.0001, *n* = 27) when approaching a patient requiring resuscitation before and after the course (Figure 3). 

**Figure 4:**
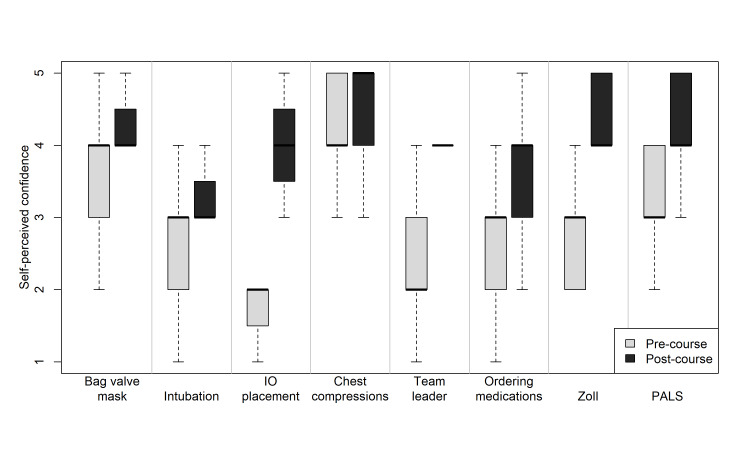
Self-perceived confidence on resuscitation skills before the course and after the course. For each skill, residents’ self-perceived confidence significantly increased from pre- to post-course (*P* < 0.01 for all), rated on a 5-point Likert scale where 1 = not at all confident and 5 = very confident.

There was also improvement in self-perceived skill ability before and after the course (Figure 4).

## Discussion

We created a novel, intensive, simulation-based rotation for pediatric residents that was feasible and well-accepted by residents but required specific resources and collaboration. Our pediatric residents found this rotation high-yield, enjoyable, and showed improvement in leadership skills and self-perceived improvement in confidence, capability, and anxiety. All components of the CALM tool improved, including leadership, communication, team management, and medical management. The new ACGME requirements have a decreased emphasis on critical care subspecialties in pediatric residency training [1]. Additionally, pediatric residents’ resuscitation skills decline within the first eight months of PALS certification [18]. A major benefit of this rotation is that it fills a clinical gap for residents who want additional experience with leadership, resuscitation management, and procedural skills. While we focused on CRM, resuscitation, and leadership skills, specific curricular content can be adapted to meet the needs of individual programs or specific residents.

There is extensive literature on the use of SBME to teach pediatric residents leadership and communication skills [9-13]. Additionally, simulation enhances pediatric residents’ retention, knowledge, and resuscitation performance [3, 6-8]. Most examples of SBME courses, such as orientation courses, workshops, and bootcamps for residents, are of shorter duration with fewer simulations than our course [19-22]. The residents in our study reported participation in SBME, but they still reported low confidence in resuscitation skills before the rotation, with improvement after course completion. While spaced repetition has demonstrated educational benefits, the massed learning approach that we utilized also proved effective in improving our residents’ leadership skills and self-assessment of resuscitation skills. The intensive nature of our course allowed residents to participate in many simulations over a short period of time, allowing them to rapidly incorporate feedback from facilitators. Additionally, the structure of the course decreased some barriers to SBME implementation among pediatric residents by simplifying scheduling between the residents, facilitators, and simulation center. A systematic review looking at spaced learning versus massed learning in resuscitation suggested, with very low certainty evidence, that spaced learning may be associated with improved educational outcomes (skill retention, skill performance) as compared to massed learning, but did not have evidence to support either type of learning about clinical outcomes [23].

An intensive rotation may not be feasible for other programs due to the barriers of creating and sustaining a course of this duration and intensity. A survey of pediatric residency program leaders demonstrated many challenges to implementing simulation, including time (facilitator and learner), physical resources (space, equipment, cost), logistics, expertise (facilitator expertise), competing priorities, and buy-in [24]. This course requires course directors who can be protected from clinical time, facilitator training, simulation equipment and mannequins, software to run high-fidelity simulations, curriculum development, agreement and support from multiple groups, recruitment of residents and interdisciplinary participants, and coordinating residency schedules. Despite the identified requirements, it is feasible to create an intensive simulation rotation for pediatric residents, as evidenced by the successful completion of six rotations and an additional occurrence after data collection. However, depending on resources and objectives of individual programs, spaced simulation may be more feasible and acceptable.

There are limitations to this study. The rotation was implemented in a single pediatric residency program and may not be generalizable to other pediatric residency programs. One limitation is that residents self-select into this course, which could bias the results of the surveys and their overall engagement. While we had a 71% response rate for the post-survey, given the small sample size, this could raise the possibility of nonresponse bias. Additionally, it is possible that a shorter rotation, for example, one week, could also be effective. While we showed improvement in self-perceived confidence, anxiety, and capability, these skills were not evaluated objectively. Much of our evaluation was based on self-assessment, and self-assessment has repeatedly been proven to be poorly correlated with other external or objective assessments of knowledge or skill [25]. Despite the limitations of self-assessment, the evaluation plan was strengthened by the external evaluation of leadership skills on a subset of participants. This course does not address retention of skills, and there is evidence that learned skills can decay over time [26]. This study does not evaluate how the rotation translates to clinical outcomes. Additionally, while course creators attempted to teach via multiple learning styles, this course focused mostly on teaching with a kinesthetic, or hands-on, approach. Some learners may benefit from a different style of teaching that better aligns with their individual learning style [27].

## Conclusions

We developed a novel, intensive, simulation-based rotation for pediatric residents that improved leadership skills and resident self-perceived levels of capability and confidence when approaching a patient resuscitation. This rotation was feasible to implement at our institution and was acceptable and well-liked by pediatric residents. Importantly, this rotation fills a gap in pediatric residency education to teach CRM skills that are not taught due to the rarity of these events and the decreasing critical care time. Next steps include additional objective skill assessments during the course, longitudinal tracking of participants to evaluate skill retention over time, and follow-up data collection on the application of learned skills in clinical practice.

## Appendices

Appendix A 

**Table 3:** Post Survey

Question								
Please enter the first two letters of their middle name, the two numbers of their month of birth, and first two numbers of their address from their childhood home. (ex: If middle name is Riley, birthday is April and childhood address is 2658, enter RI0426)								
Please select your level of training.								
	PGY2							
	PGY3							
What field of pediatrics are you interested in?								
	Adolescent Medicine							
	Cardiology							
	Child Abuse Pediatrics							
	Development-Behavioral Pediatrics							
	Emergency Medicine							
	Endocrine							
	Gastroenterology							
	Hematology/Oncology							
	Hospital Medicine							
	Infectious Disease							
	Nephrology							
	Neurology							
	Neonatology (NICU)							
	Pediatric Critical Care (PICU)							
	Primary Care							
	Pulmonology							
	Rheumatology							
	Sports Medicine							
	Other							
During this rotation, how often did you perform the following roles?		0	1	2	3	4	5	>5
	Bag-mask ventilation							
	Intubation							
	IV placement							
	IO placement							
	Chest compressions							
	Team leader							
	Recorder							
	Ordering medications							
	Drawing up medications							
	Use of zoll defibrillator							
	Use of PALS algorithm							
	Observer							
After this rotation, how confident do you feel in the following skills as a provider in a resuscitation?								
		Not at all confident -1	2	3	4	Very confident -5		
	Bag-mask ventilation							
	Intubation							
	IV placement							
	IO placement							
	Chest compressions							
	Team leader							
	Recorder							
	Ordering medications							
	Drawing up medications							
	Use of zoll defibrillator							
	Use of PALS algorithm							
	Observer							
Please rate your level of anxiety when you approach a patient needing resuscitation.								
	Not at all anxious							
	A little anxious							
	Somewhat anxious							
	Pretty anxious							
	Extremely anxious							
How capable do you feel running a code?								
	Not at all capable							
	A little capable							
	Somewhat capable							
	Pretty capable							
	Extremely capable							
How confident do you feel in leading a code?								
	Not at all confident							
	A little confident							
	Somewhat confident							
	Pretty confident							
	Extremely confident							
How comfortable do you feel with your level of knowledge on how to manage patients needing resuscitation?								
	Not at all comfortable							
	A little comfortable							
	Somewhat comfortable							
	Pretty comfortable							
	Extremely comfortable							
Did you like the simulation rotation?								
	Did not like it at all							
	Liked it a little							
	Somewhat liked it							
	Liked it a lot							
	Loved it							
Are you glad you took the simulation course?								
	Not at all glad							
	A little glad							
	Somewhat glad							
	Pretty glad							
	Extremely glad							
Were you satisfied with the amount of simulation during this rotation?								
	Not at all satisfied							
	A little satisfied							
	Somewhat satisfied							
	Pretty satisfied							
	Very satisfied							
Were you satisfied with how many simulations you were able to lead in this rotation?								
	Not at all satisfied							
	A little satisfied							
	Somewhat satisfied							
	Pretty satisfied							
	Very satisfied							
Comment on the advantages and disadvantages of doing the simulations at the CHCO Simulation Center.								
Comment on the advantages and disadvantages of doing the simulations at Denver Health.								
Were you satisfied with the content that was covered in this course?								
	Not at all satisfied							
	A little satisfied							
	Somewhat satisfied							
	Pretty satisfied							
	Very satisfied							
What content was not covered that you think is important?								
Would you recommend this course to your co-residents?								
	Not at all recommend							
	Maybe recommend							
	Probably recommend							
	Definitely recommend							
What did you learn that you will take with you to the bedside?								
Please provide any additional comments about the simulation rotation below.								

Appendix B 

**Table 4:** Pre Survey

Question									
Please enter the first two letters of their middle name, the two numbers of their month of birth, and first two numbers of their address from their childhood home. (ex: If middle name is Riley, birthday is April and childhood address is 2658, enter RI0426)									
Please select your level of training.									
	PGY2								
	PGY3								
What field of pediatrics are you interested in?									
	Adolescent Medicine								
	Cardiology								
	Child Abuse Pediatrics								
	Development-Behavioral Pediatrics								
	Emergency Medicine								
	Endocrine								
	Gastroenterology								
	Hematology/Oncology								
	Hospital Medicine								
	Infectious Disease								
	Nephrology								
	Neurology								
	Neonatology (NICU)								
	Pediatric Critical Care (PICU)								
	Primary Care								
	Pulmonology								
	Rheumatology								
	Sports Medicine								
	Other								
How many actual resuscitations have you been to in residency (excluding standard NICU/WBN delivery resuscitations)?									
	0								
	1-2								
	3-5								
	6-9								
	>9								
In how many actual resuscitations did you play an active role, i.e. stabilizing the airway, performing CPR, being team leader, IV/IO placement, recorder? Select N/A if you answered zero to the previous question.									
	0								
	1-2								
	3-5								
	6-9								
	>9								
	N/A								
How did you participate? Please identify for each of the following activities how often you performed each of them in active resuscitations?		N/A	0	1	2	3	4	5	>5
	Bag-mask ventilation								
	Intubation								
	IV placement								
	IO placement								
	Chest compressions								
	Team leader								
	Recorder								
	Ordering medications								
	Drawing up medications								
	Use of zoll defibrillator								
	Use of PALS algorithm								
	Observer								
In how many actual resuscitations were you the first provider on scene?									
	0								
	1-2								
	3-5								
	6-9								
	>9								
How many simulated pediatric resuscitations have you participated in during residency?									
	0								
	1-2								
	3-5								
	6-9								
	>9								
How many interdisciplinary simulations have you participated in during residency?									
	0								
	1-2								
	3-5								
	6-9								
	>9								
How did you participate in simulated pediatric resuscitations? Please identify for each of the following activities how often you performed each of them in active resuscitations.		0	1	2	3	4	5	>5	
	Bag-mask ventilation								
	Intubation								
	IV placement								
	IO placement								
	Chest compressions								
	Team leader								
	Recorder								
	Ordering medications								
	Drawing up medications								
	Use of zoll defibrillator								
	Use of PALS algorithm								
	Observer								
What type of simulated resuscitation activities have you participated in during residency? Please check all that apply.									
	Simulated resuscitations in high fidelity simulation center								
	Simulated resuscitations in low fidelity, non-patient environment (i.e. classroom)								
	Simulated resuscitations in low fidelity, patient-centered environment (i.e. at bedside, empty patient room)								
	Procedure training (i.e. bag-mask ventilation, intubation, placeing IOs, drawing epi)								
	Simulated resuscitations that are high fidelity, in a patient-centered environment (ex: Denver Health)								
	Interdisciplinary simulations (ex: with nursing, RT, pharmacy, etc)								
Of the above that you have participated in, how effective is simulation in building your skills or knowledge as a provider in resuscitations? (LEAVE blank if you have not participated).		Not at all effective	A little effective	Somewhat effective	Pretty effective	Very effective			
	Low fidelity simulation								
	High fidelity simulation								
	Procedural training (ex. intubation, lumbar puncture, etc)								
How many second year PICU rotations have you completed in the ICU at Children's Hospital Colorado?									
	0								
	1								
	2								
How many mock codes or simulations have you done in the PICU at Children's?									
	0								
	1-2								
	3-5								
	>5								
	N/A if haven't done PICU at CHCO								
How important do you feel these skills are in training in pediatrics?		Not at all important -1	2	3	4	Very important -5			
	Bag-mask ventilation								
	Intubation								
	IV placement								
	IO placement								
	Chest compressions								
	Team leader								
	Recorder								
	Ordering medications								
	Drawing up medications								
	Use of zoll defibrillator								
	Use of PALS algorithm								
	Observer								
How confident do you feel in the following skills as a provider in a resuscitation?		Not at all confident -1	2	3	4	Very confident -5			
	Bag-mask ventilation								
	Intubation								
	IV placement								
	IO placement								
	Chest compressions								
	Team leader								
	Recorder								
	Ordering medications								
	Drawing up medications								
	Use of zoll defibrillator								
	Use of PALS algorithm								
	Observer								
Please rate your level of anxiety when you approach a patient needing resuscitation.									
	Not at all anxious								
	A little anxious								
	Somewhat anxious								
	Pretty anxious								
	Extremely anxious								
How capable do you feel running a code?									
	Not at all capable								
	A little capable								
	Somewhat capable								
	Pretty capable								
	Extremely capable								
How confident do you feel in leading a code?									
	Not at all confident								
	A little confident								
	Somewhat confident								
	Pretty confident								
	Extremely confident								
How comfortable do you feel with your level of knowledge on how to manage patients needing resuscitation?									
	Not at all comfortable								
	A little comfortable								
	Somewhat comfortable								
	Pretty comfortable								
	Extremely comfortable								
How important is it that you receive training using simulation modalities (low fi, high fi, procedural) in order to prepare you to lead resuscitations and have competency in many of the skills listed in the above prompts (bag-mask ventilation, chest compressions, intubation, use of the zoll, use of PALS algorithms)?									
	Not at all important								
	A little important								
	Somewhat important								
	Pretty important								
	Very important								
